# DLG1-AS1 is activated by MYC and drives the proliferation and migration of hepatocellular carcinoma cells through miR-497-5p/SSRP1 axis

**DOI:** 10.1186/s12935-020-01667-0

**Published:** 2021-01-06

**Authors:** Jie Min, Dayong Jin, Feng Zhang, Yanxia Kang, Yuhong Qi, Pang Du

**Affiliations:** 1 Department of Oncology, Tangdu Hospital, Xi’an, 710038 Shaanxi China; 2 Department of Radiology, Tangdu Hospital, Xi’an, 710038 Shaanxi China; 3 Department of Radiotherapy, Tangdu Hospital, Xi’an, 710038 Shaanxi China

**Keywords:** DLG1-AS1, miR-497-5p, MYC, SSRP1, Hepatocellular carcinoma

## Abstract

**Background:**

Long non-coding RNAs (lncRNAs) have been reported to be biological regulators in hepatocellular carcinoma (HCC). DLG1 antisense RNA 1 (DLG1-AS1) has been found to be up-regulated in cervical cancer. However, its function and underlying mechanism in HCC remains unknown.

**Methods:**

DLG1-AS1 expression was assessed in HCC cells and normal cell by RT-qPCR. Luciferase reporter assay, RNA pull down assay and RIP assay were used to demonstrate the interaction between DLG1-AS1 and miR-497-5p.

**Results:**

DLG1-AS1 was highly expressed in HCC cells. Silencing of DLG1-AS1 led to the inhibition of HCC cell growth and migration. Besides, MYC induced the transcriptional activation of DLG1-AS1. MYC could facilitate HCC cellular processes by up-regulating DLG1-AS1. MiR-497-5p could interact with DLG1-AS1 in HCC cells. Down-regulation of miR-497-5p could reverse the impacts of DLG1-AS1 silencing on HCC cells. SSRP1 expression could be positively regulated by DLG1-AS1 but was negatively regulated by miR-497-5p. Knockdown of DLG1-AS1 suppressed tumor growth in nude mice.

**Conclusions:**

DLG1-AS1 is activated by MYC and functions as an oncogene in HCC via miR-497-5p/SSRP1 axis. 
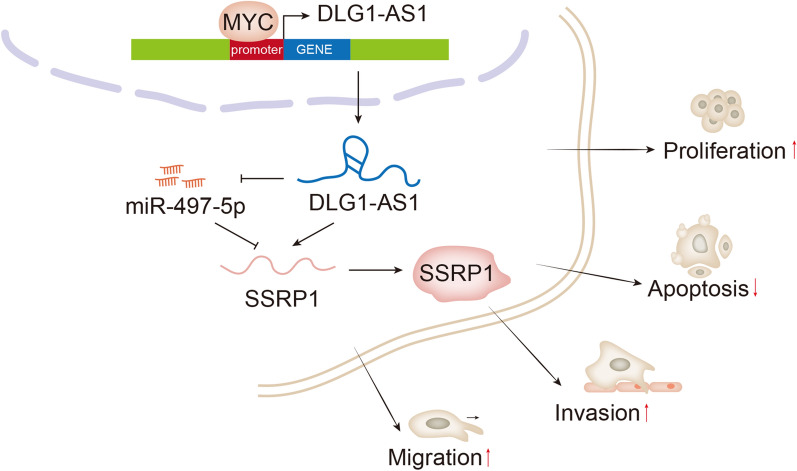

## Background

Hepatocellular carcinoma (HCC) is one of the most aggressive and fatal cancers among human beings all over the world, which is hard to be cured due to metastasis [1]. The common treatments for HCC such as surgery, chemotherapy, radiotherapy and biotherapy always had limited effects [2]. In recent years, more and more attention has been focused on targeted therapy [3]. Although the effects of identified targets are not full-scaled applied in clinical practice, the potential value of these targets is promising [4]. Thus, it is necessary to unveil the underlying molecular mechanism in HCC progression to find novel effective therapeutic targets.

Long non-coding RNAs (lncRNAs) belong to non-coding RNAs, which have no or limited protein-coding capacities [5]. Emerging studies have reported that lncRNAs are crucial regulators in various disease processes [6–8]. For example, lncRNA GHET1 is a biomarker in HCC and accelerates tumor progression by targeting KLF2 [9]. HOXA11-AS facilitates the proliferation of HCC cells [10]. DLG1 antisense RNA 1 (DLG1-AS1) has been identified as a novel oncogenic lncRNA in cervical cancer [11]. Recently, it has been proven to be a tumor promoter in breast cancer by negatively regulating miR-203 [12]. However, the role of DLG1-AS1 in HCC has not been well explained.

It has been widely reported that transcription factors can bind to the promoter region of lncRNAs, thus inducing the transcriptional activation and upregulation of lncRNAs in human cancers [13–16]. MYC transcription factor has been demonstrated to be an transcription activator for several lncRNAs. For example, MYC can bind to LINC00346 promoter to activate LINC00346 transcription [17]. MYC can trans-activate HOXC-AS1 and form a positive feedback loop with HOXC-AS1 in gastric cancer [18]. However, it is unclear whether MYC can act as a positive upstream regulator for DLG1-AS1.

MicroRNAs (miRNAs) exert crucial functions in modulating progression of multiple cancers, including HCC [19]. For instance, miR-3117 contributes to HCC progression via targeting PHLPPL [20]. MiR-122 suppressed EMT in HCC through targeting Snail1/2 and inactivating WNT/β-catenin pathway [21]. Competing endogenous RNA (ceRNA) has been recognized as a crucial post-transcriptional regulatory mechanism involved in the development of various human cancers [22, 23]. In a ceRNA pathway, miRNAs can interact with lncRNAs to regulate their downstream mRNAs in tumor progression. For example, HCAL works as a ceRNA of LAPTM4B to boost the proliferation and motility in HCC [24]. CASC2 exerts inhibitory function on epithelial-mesenchymal transition (EMT) of HCC by targeting miR-367/FBXW7 [25]. LncRNA DSCR8 accelerates HCC progression and activates Wnt/β-catenin pathway in HCC through sponging miR-485-5p [26]. This study was aimed at investigating the miRNA interacting with DLG1-AS1 and thus unveiling a novel ceRNA pathway.

In summary, the main purpose of our study was to uncover the function of DLG1-AS1 in HCC and explore its underlying molecular mechanism.

## Methods

### Cell culture

Human HCC cell lines (MHCC97-L, Huh-7, Hep3B and HCCLM3) and the normal human liver immortalized cells (THLE-2) were obtained from ATCC (Rockville, Maryland) and were propagated under 5% CO_2_ and 37 °C. DMEM with 10% FBS (Invitrogen) as supplement was available from Invitrogen (Carlsbad, CA) for cell culture.

### RNA extraction and RT-qPCR

Total RNA was extracted from cells with Trizol kit as per the standard protocol (Invitrogen). cDNA template was synthesized based on the instruction of reverse transcription kit (Applied Biosystems, Carlsbad, CA). To assess gene expression, qPCR was implemented based on the protocol of StepOne™ Real-Time PCR System (Applied Biosystems). All data were calculated using 2^−ΔΔCt^ method with U6 or GAPDH as the internal control.

### Transfection

shRNAs and control shRNAs were designed and procured from RiboBio (Guangzhou, China) for silencing of DLG1-AS1, FOS, STAT1, MYC or E2F6. For overexpression of knockdown of miR-497-5p, miR-497-5p mimics and NC mimics, miR-497-5p inhibitor and NC inhibitor were synthesized by Genepharma (Shanghai, China). Similarly, pcDNA3.1/DLG1-AS1, pcDNA3.1/SSRP1 and pcDNA3.1 empty vector were also synthesized for overexpression. Transfections were finished by use of Lipofectamine2000 (Invitrogen). At 48 h post-transfection, cells were reaped.

### Colony formation assay

Hep3B and HCCLM3 cells were seeded into 6-well plates at a density of 500 cells per well and cultured for 14 days. After fixed with methanol, colonies were dyed in 1% crystal violet solution for visualization and counting.

### EdU assay

Cell proliferation was also detected by EdU assay as per manual (Click-iT® EdU Imaging Kits; Invitrogen). Cells were planted in 96-well plates at 8 × 10^3^ cells per well and incubated all night. Afterwards, cells were incubated with EdU medium for 4 h. Cell nuclei were processed with DAPI dye and imaged with fluorescent microscope.

### Flow cytometry assay

Annexin V-FITC/PI staining was applied to detect the apoptotic cells. After double-staining as per direction, 1 × 10^5^ cell samples were assayed by FACSCalibur flow cytometer (BD Biosciences, San Jose, CA, USA).

### TUNEL assay

Transfected cells were first fixed and permeabilized, following washing in PBS. Cells were then cultivated under 37 °C with 50 μl of TUNEL reaction buffer for as required by manual of TUNEL staining kit (Beyotime, Shanghai, China). DAPI staining was performed before analysis by fluorescent microscope.

### Transwell assays

Migration assay was performed by using Transwell chambers (Corning, Corning, NY). The lower chamber filled with complete medium. Cells suspended in serum-free medium were put into upper chamber for 24 h. Cells on the bottom surface were counted using 0.1% crystal violet dye and microscope. Invasion assay was conducted using transwell chambers coated with Matrigel.

### Chromatin immunoprecipitation (ChIP) assay

ChIP assay was achieved with ChIP kit (Millipore, Billerica, MA) as per standard method. After obtaining the DNA and protein cross-linking, DNA was fragmented by ultrasonic for immunoprecipitation with anti-MYC or control anti-IgG antibody (Millipore). Relative RNA enrichments were assayed by qPCR.

### Luciferase reporter assay

Cells in 24-well plates were co-transfected with pGL3-DLG1-AS1 promoter-WT/Mut, pRL-TK-Renilla and sh-MYC/sh-NC. Besides, pmirGLO-DLG1-AS1-WT/Mut and pmirGLO-SSRP1-WT/Mut reporter vectors were severally co-transfected with miR-497-5p mimics or NC mimics into cells. Luciferase assays were achieved with Luciferase Reporter Assay System (Promega, Madison, WI) in line with instruction.

### FISH

The RNA FISH probe for DLG1-AS1 was available from Ribobio and used as per manufacturer’s protocol. Cell nuclei were detected via Hoechst staining and captured by fluorescent microscope.

### RNA pull down assay

Protein extracts from cells were cultivated with the wild-type or mutant biotinylated miR-497-5p probes, as well as streptavidin agarose magnetic beads. RT-qPCR was conducted to evaluate RNA enrichment.

### RNA immunoprecipitation (RIP) assay

RIP assay were carried out using Magna RIP™ RNA-Binding Protein Immunoprecipitation Kit (Millipore) and human Ago2 antibody as per standard protocol. IgG antibody was used as negative control. After immunoprecipitation, RT-qPCR was performed for evaluation.

### Western blot

Protein extracts were separated by 10% SDS-PAGE, shifted to PVDF membranes and mounted in 5% nonfat milk for 1 h. Primary antibodies against control GAPDH and SSRP1, along with the HRP-tagged secondary antibodies were all available from Abcam (Cambridge, MA). Following washing in TBST, protein blots were visualized by ECL Substrates (Millipore).

### Subcutaneous xenograft experiment

Male BALB/C nude mice (6 weeks old) were available from Beijing Vital River Laboratory Animal Technology Co. Ltd. (Beijing, China) and employed with the approval from the Animal Research Ethics Committee of Tangdu Hospital. HCCLM3 cells were injected subcutaneously to nude mice at a density of 1 × 10^6^ cells. Tumor volume was monitored every 4 days. Mice were killed at 28-days’ post-injection, tumors were carefully dissected and then weighed.

### Immunohistochemistry (IHC)

IHC assay was implemented using the paraffin-embedded tumor tissue samples from nude mice. The paraformaldehyde-fixed paraffin sections were prepared to incubate with anti-Ki67 and anti-PCNA (Abcam). Sections were visualized under a microscope.

### Statistical analyses

Averages of three independent experimental obtained and the results were exhibited with mean ± Standard Deviation (S.D.). Statistical analyses were conducted with Prism 5.0 software (GraphPad Software, Inc., La Jolla, CA) using Student’s t-test and one-way ANOVA. Significant values were specified as p < 0.05.

## Results

### DLG1-AS1 promotes the growth and migration of HCC cells

**Fig. 1:**
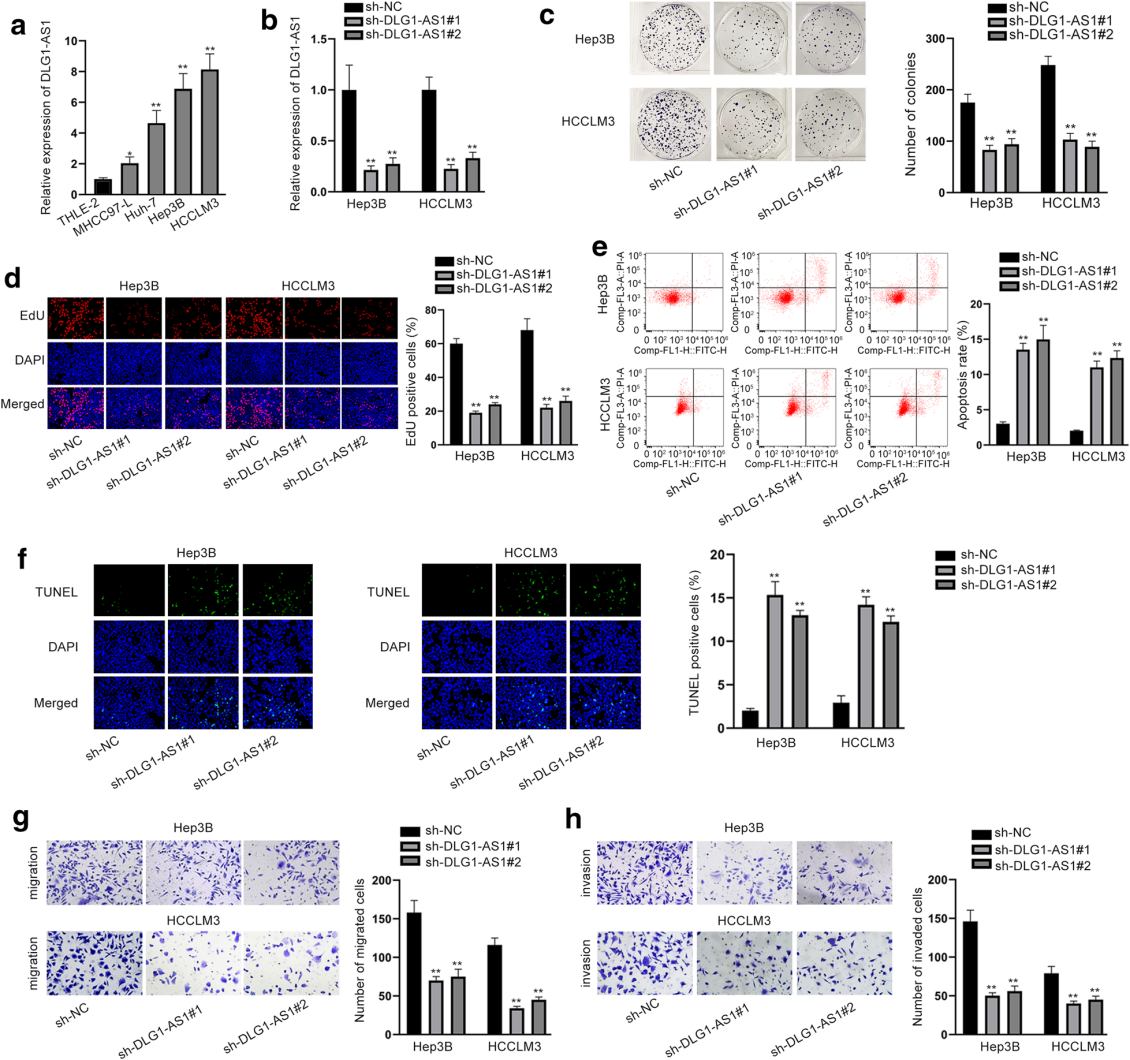
DLG1-AS1 promotes HCC cell growth and migration. **a** The expression of DLG1-AS1 was tested in HCC cell lines (MHCC97-L, Huh-7, Hep3B and HCCLM3) and in the normal Human liver immortalized cell line (THLE-2). **b** The efficiency of DLG1-AS1 knockdown was analyzed via RT-qPCR in Hep3B and HCCLM3 cells. **c**, **d** The proliferative ability of Hep3B and HCCLM3 cells transfected with sh-DLG1-AS1#1/2 or sh-NC was evaluated via colony formation and EdU assays. **e**, **f** Cell apoptosis rate in different groups was analyzed by TUNEL assay and flow cytometry analysis. **g**, **h** Transwell assays were performed to measure the capabilities of migration and invasion. ^*^P < 0.05, ^**^P < 0.01

Data of RT-qPCR revealed that DLG1-AS1 was expressed higher in HCC cell lines (MHCC97-L, Huh-7, Hep3B and HCCLM3) than that in the normal human liver immortalized cell line (THLE-2) (Fig. 1a). Since Hep3B and HCCLM3 possessed highest expression of DLG1-AS1 in HCC cells, they were used in the following assays. For loss-of function assays, DLG1-AS1 was silenced by transfecting sh-DLG1-AS1#1/2 into Hep3B and HCCLM3 cells (Fig. 1b). The results of EdU and colony formation assays revealed that down-regulation of DLG1-AS1 repressed the proliferative abilities of HCC cells (Fig. 1c, d). The protein levels of PCNA, CDK1 and cyclin D1 were all decreased by DLG1-AS1 silencing (Additional file 1: Fig. S1a). Meanwhile, down-regulated DLG1-AS1 increased apoptosis rate (Fig. 1e, f), which was further strengthened by the changes in apoptosis-related proteins (Additional file 1: Fig. S1b). Additionally, cell migration and invasion were reduced by the knockdown of DLG1-AS1 (Fig. 1g, h). To analyze whether DLG1-AS1 exerted functions through signaling pathways, we detected the levels of proteins associated with AKT/mTOR and Src/FAK signaling pathways. As shown in Additional file2: Fig. S2a, b, the levels of p-AKT, p-mTOR, p-Src and p-FAK were all decreased after silencing of DLG1-AS1. We also overexpressed DLG1-AS1 in normal THLE-2 cells (Additional file 3: Fig. S3a) and conducted functional assays. Overexpression of DLG1-AS1 promoted cell proliferation but the suppressed apoptosis (Additional file 3: Fig. S3b, c and Fig. S3d, f). The migration and invasion were also facilitated by the overexpression of DLG1-AS1 (Additional file 3: Fig. S3g, h). Importantly, high level of DLG1-AS1 increased the levels of p-AKT, p-mTOR, p-Src and p-FAK (Additional file 3: Fig. S3i, j). In summary, DLG1-AS1 is up-regulated in HCC cells and promotes cellular processes.

### MYC induces the transcriptional activation of DLG1-AS1 to boost HCC cell proliferation and migration

**Fig. 2:**
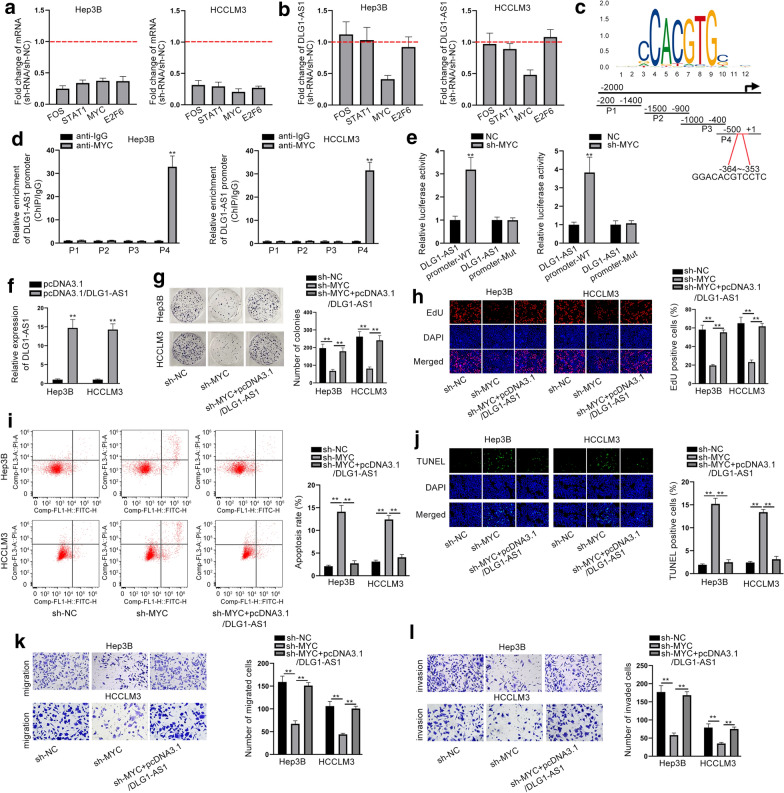
MYC activates DLG1-AS1 transcription to boost HCC cell proliferation and migration. **a** RT-qPCR measured mRNAs expression of transcription factor. **b** DLG1-AS1 expression was detected by knockdown of these expressions of transcription factor in RT-qPCR. **c** DNA motif of MYC and its binding sites in DLG1-AS1 promoter. **d** The enrichment of four parts of DLG1-AS1 promoter in anti-MYC group and anti-IgG group. **e** Luciferase reporter assay demonstrated the relationship between DLG1-AS1 promoter and MYC. **f** RT-qPCR assessed DLG1-AS1 expression in cells transfected with pcDNA3.1/DLG1-AS1. **g**, **h** Colony formation and EdU assays detected proliferation in Hep3B and HCCLM3 cells transfected with sh-NC, sh-MYC and pcDNA3.1/DLG1-AS1. **i**, **j** Apoptosis was examined in different group by flow cytometry and TUNEL. **k**, **l** Migration and invasion were evaluated in transwell among different group. ^**^P < 0.01

We continued to explore the upstream mechanism of DLG1-AS1 in HCC. According to UCSC (http://genome.ucsc.edu/), we discovered that FOS, STAT1, MYC and E2F6 were the potential transcription factors of DLG1-AS1. Then, we silenced them and evaluated their knockdown efficiency using RT-qPCR (Fig. 2a). The protein level of MYC was also examined and shown in Additional file 4: Fig. S4a. Then, we uncovered that only MYC knockdown could lessen the expression of DLG1-AS1 (Fig. 2b). Likewise, silencing of DLG1-AS1 decreased the levels of MYC mRNA and protein (Additional file 4: Fig. S4b, c). Next, the DNA motif of MYC (Fig. 2c) and its binding region in DLG1-AS1 promoter were obtained from JASPAR (http://jaspar.genereg.net/). We divided the upstream 2000 bases of DLG1-AS1 promoter into four pieces and named them as P1, P2, P3 and P4 (Fig. 2c). ChIP assays disclosed that only P4 could bind to YY1 (Fig. 2d). Luciferase reporter assay demonstrated that DLG1-AS1 promoter activity could be reduced by the knockdown of MYC (Fig. 2e). Next, we elevated DLG1-AS1 expression (Fig. 2f). It was found that down-regulated MYC suppressed proliferation but overexpression of DLG1-AS1 reversed this tendency (Fig. 2g, h). Meanwhile, the levels of PCNA, CDK1 and Cyclin D1 were decreased by MYC silencing, but were enhanced again by the overexpression of DLG1-AS1 (Additional file 5: Fig. S5a). Additionally, the ascending tendency of apoptosis caused by MYC knockdown was restored by the up-regulation of DLG1-AS1 (Fig. 2i, j and Additional file 5: Fig. S5b). Suppression of migration and invasion caused by silenced MYC was recovered by DLG1-AS1 up-regulation (Fig. 2k, l). The inhibitory effects of MYC silencing on the signals of AKT/mTOR pathway and Src/FAK pathway were attenuated by DLG1-AS1 overexpression (Additional file 5: Fig. S5c, d). Additionally, we repeated all experiments in HCC cells transfected with sh-NC, sh-DLG1-AS1#1 and sh-DLG1-AS1#1 + pcDNA3.1/MYC. As a result, MYC overexpression could also attenuate the effects of DLG1-AS1 silencing on cellular processes and pathways (Additional file 6: Fig. S6a–j). All in all, MYC induces the transcriptional activation of DLG1-AS1 to strengthen HCC cellular processes.

### DLG1-AS1 boosts proliferation and migration of HCC cells via sponging miR-497-5p

**Fig. 3:**
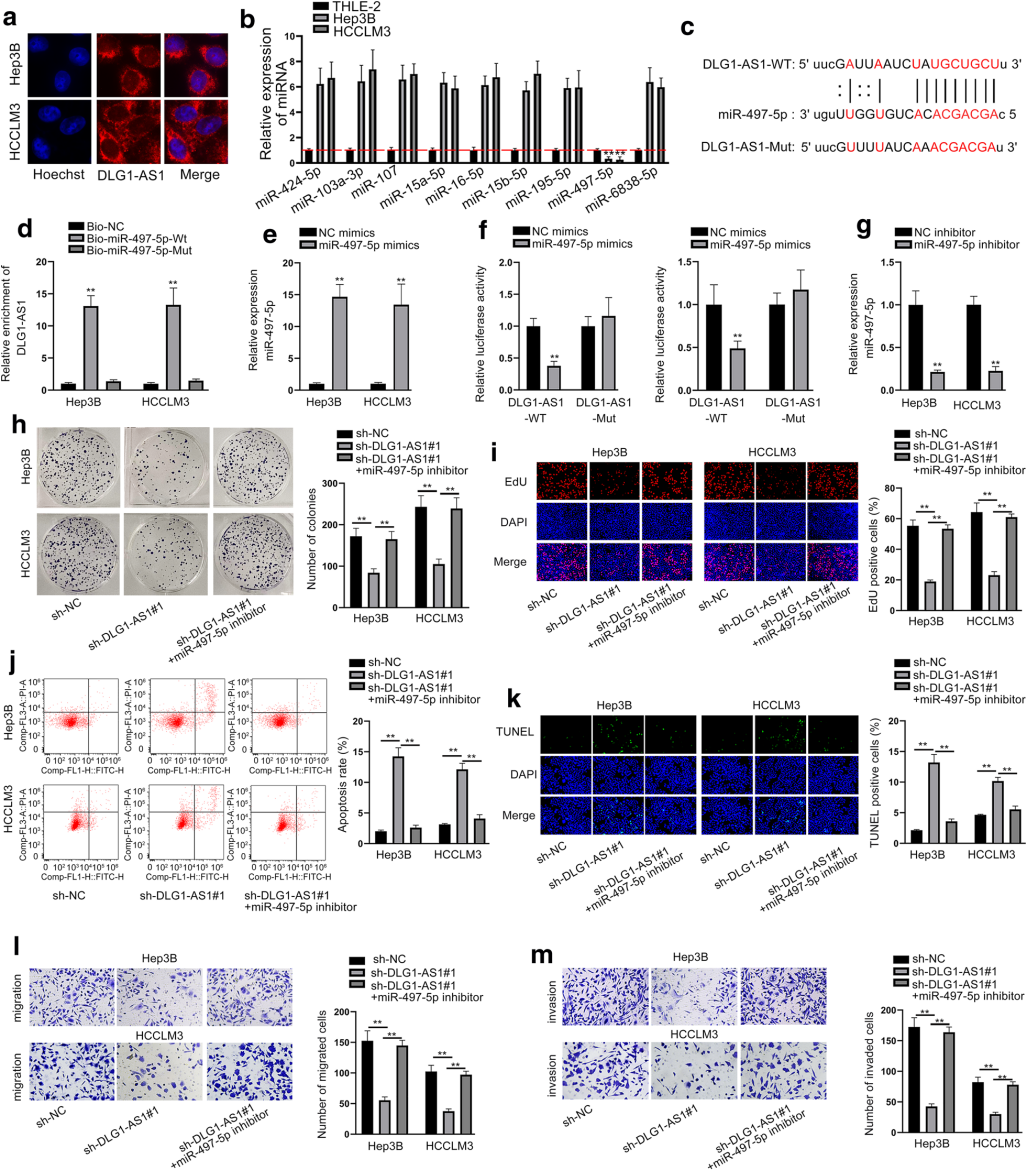
DLG1-AS1 boosts the proliferation and migration of HCC cells via sponging miR-497-5p. **a** FISH assay was applied to ascertain the cellular distribution of DLG1-AS1 in Hep3B and HCCLM3 cells. **b** Expression of 9 miRNAs in HCC cells and THLE-2 was detected by RT-qPCR. **c** A binding site between DLG1-AS1 and miR-497-5p. **d** RNA pull-down illustrated miR-497-5p bound to DLG1-AS1. **e** The efficiency of miR-497-5p overexpression in Hep3B and HCCLM3 cells. **f** Luciferase reporter assay demonstrated the relationship between DLG1-AS1 and miR-497-5p. **g** MiR-497-5p expression was examined by RT-qPCR in cells transfected with miR-497-5p inhibitor. **h**–**m** Cell proliferation, apoptosis, migration and invasion were appraised in cells transfected with sh-NC, sh-DLG1-AS1#1 and miR-497-5p inhibitor. ^**^P < 0.01

Then, we investigated the downstream mechanism of DLG1-AS1. To begin with, FISH assay was performed to identify the subcellular location of DLG1-AS1. Result showed that DLG1-AS1 mainly located in the cytoplasm (Fig. 3a). Subsequently, we used starBase (http://starbase.sysu.edu.cn) to select out 9 miRNAs possessing complementary base paring with DLG1-AS1. Through RT-qPCR analysis, only miR-497-5p was down-regulated in HCC cells (Fig. 3b). The binding sequences between DLG1-AS1 and miR-497-5p were shown in Fig. 3c. RNA pull down assay revealed that DLG1-AS1 was enriched in biotinylated miR-497-5p-Wt group (Fig. 3d). Then, we overexpressed miR-497-5p by transfecting miR-497-5p mimics into HCC cells (Fig. 3e). The data of luciferase reporter assay disclosed that miR-497-5p up-regulation cut down the luciferase activity of DLG1-AS1-WT (Fig. 3f). Rescue assays were performed for a better demonstration. First, miR-497-5p expression was diminished by miR-497-5p inhibitor (Fig. 3g). The results of rescue assays presented that the effects of down-regulated DLG1-AS1 on proliferation, apoptosis, migration and invasion were countervailed by the depletion of miR-497-5p (Fig. 3h–m and Additional file 7: Fig. S7a, b). Additionally, inhibition of miR-497-5p promoted HCC cell proliferation, migration and invasion (Additional file 8: Fig. S8a–d). Altogether, DLG1-AS1 promotes the cellular processes in HCC by interacting with miR-497-5p.

### DLG1-AS1 works as a miR-497-5p sponge to modulate SSRP1 expression in HCC cells

**Fig. 4:**
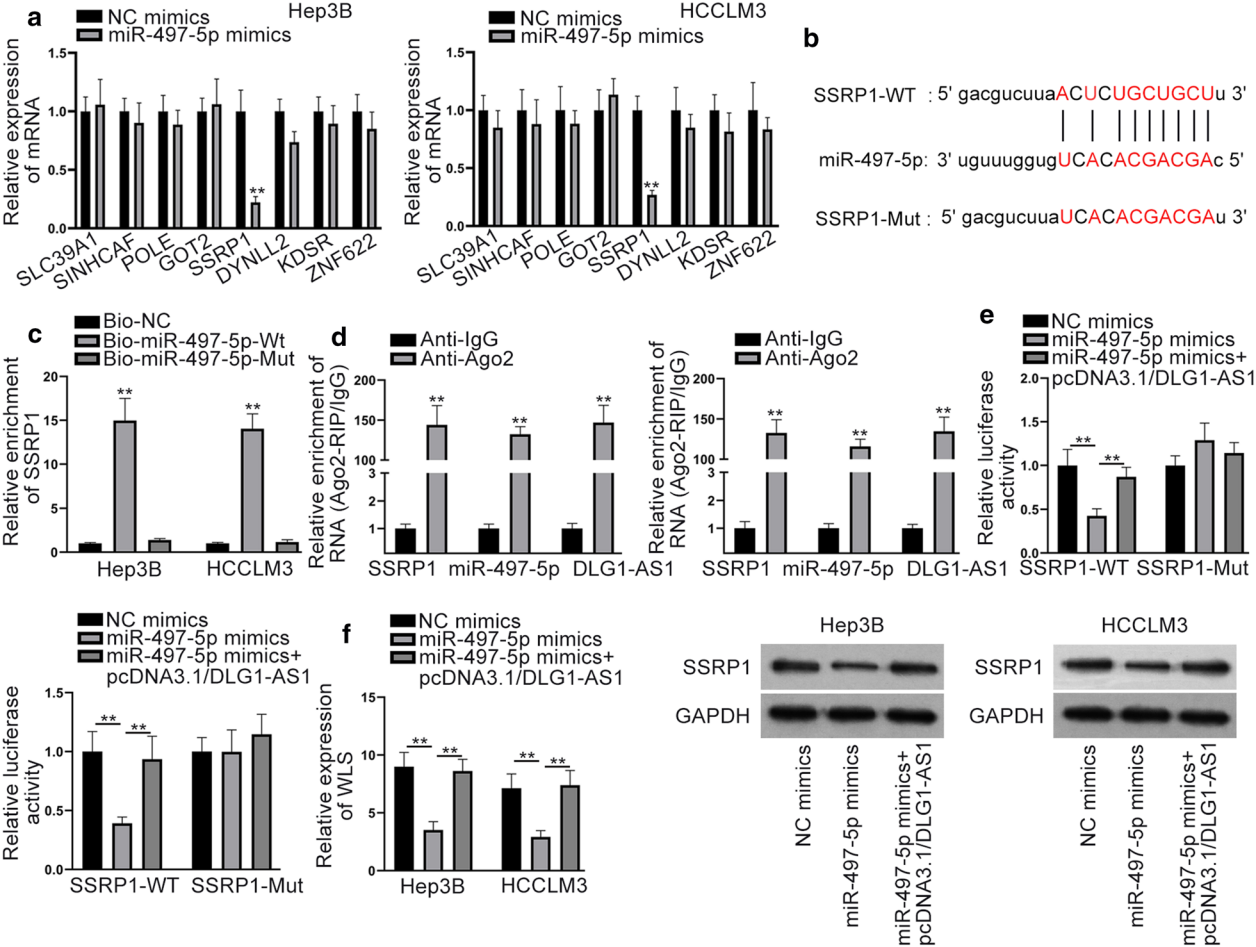
DLG1-AS1 works as a miR-497-5p sponge to modulate SSRP1 in HCC cells. **a** RT-qPCR detected 8 mRNAs expression in cell transfected with miR-497-5p mimics. **b** Bioinformatics predicted binding sequence of miR-497-5p and SSRP1. **c** RNA pull down certified miR-497-5p could bind to SSRP1. **d** RIP assay validated SSRP1, DLG1-AS1 and miR-497-5p coexisted in RISC. **e** Luciferase reporter assay verified the competing relationship among DLG1-AS1 and SSRP1. **f** RT-qPCR and western blot were used to measure mRNA and protein levels of SSRP1 in cells transfected with NC mimics, miR-497-5p mimics and miR-497-5p mimics + pcDNA3.1/DLG1-AS1. ^**^P < 0.01

We continually explored the downstream targets of miR-497-5p in HCC. StarBase predicted 8 mRNAs could bind to miR-497-5p [CLIP: strict stringency (> = 5), Degradome: high stringency (> = 3)] By transfecting miR-497-5p mimics into HCC cells, we observed that only SSRP1 was down-regulated (Fig. 4a). Thus, SSRP1 was chosen for subsequent analyses. The binding sequence between miR-497-5p and SSRP1 was shown in Fig. 4b. RNA pull down assay confirmed that miR-497-5p could bind to SSRP1 (Fig. 4c). RT-qPCR analysis showed that SSRP1, miR-497-5p and DLG1-AS1 were all precipitated by Ago2 antibody (Fig. 4d). Luciferase reporter assay manifested that luciferase activity of SSRP1-WT was decreased by up-regulation of miR-497-5p. Meanwhile, overexpression of DLG1-AS1 augmented the luciferase activity of SSRP1-WT (Fig. 4e). Moreover, mRNA and protein levels of SSRP1 dropped by miR-497-5p up-regulation were enhanced by DLG1-AS1 overexpression (Fig. 4f). Taken together, SSRP1 was the downstream target of miR-497-5p.

### DLG1-AS1 contributes to cellular processes by upregulating SSRP1

**Fig. 5:**
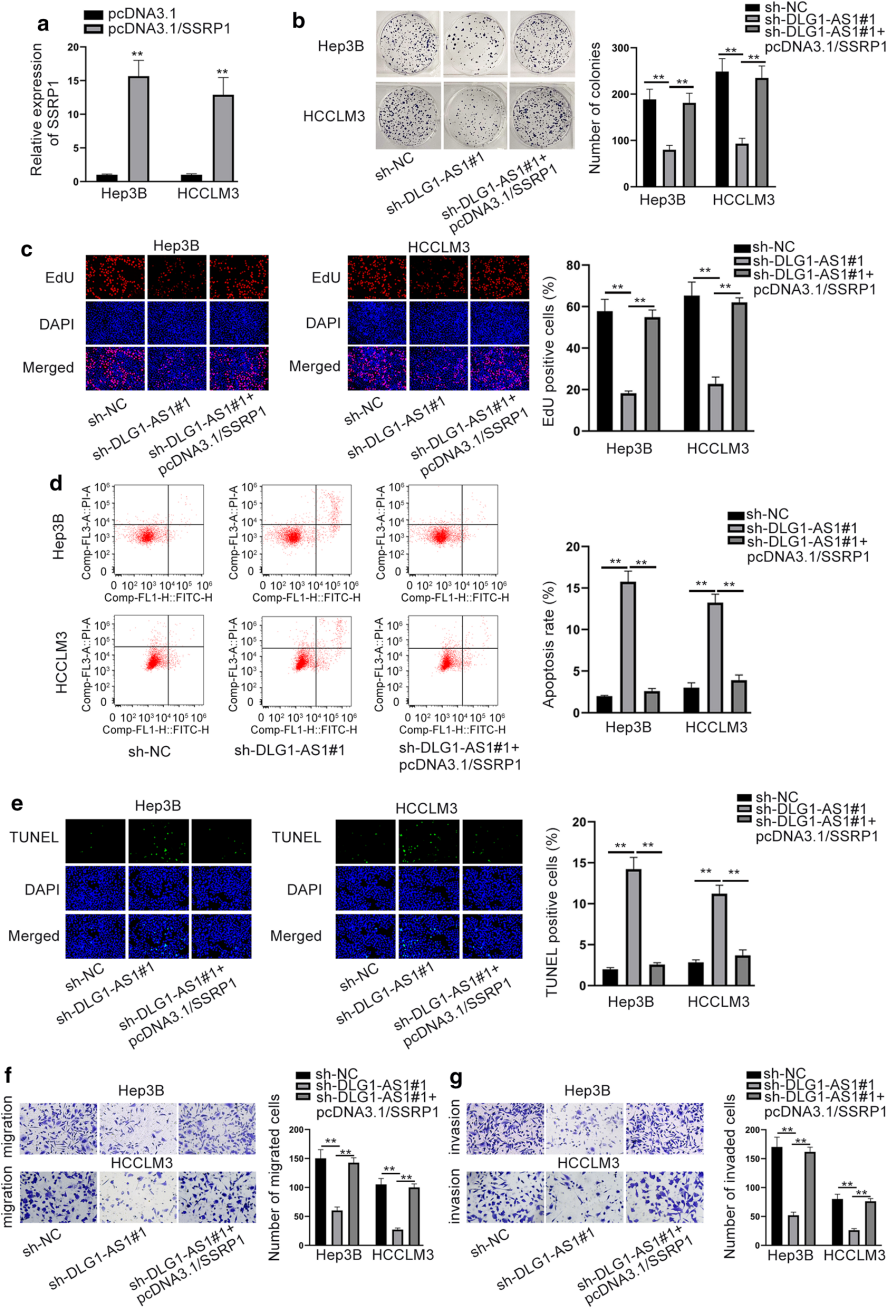
DLG1-AS1 contributes to cellular processes in HCC by upregulating SSRP1. **a** SSRP1 expression in transfected cells was detected via RT-qPCR. **b**, **c** The proliferation of transfected cells was evaluated via colony formation and EdU. **d**, **e** Cell apoptosis rate in sh-NC, sh-DLG1-AS1#1 and pcDNA3.1/SSRP1 group was evaluated by flow cytometry analysis and TUNEL. **f**, **g** Transwell was utilized to measure the capabilities of transfected cells to migrate and invade. ^**^P < 0.01

Next, we analyzed whether DLG1-AS1 aggravated HCC by regulating SSRP1. We firstly increased the expression of DLG1-AS1 by transfecting pcDNA3.1/SSRP1 into HCC cells (Fig. 5a). As shown in colony formation and EdU assays, the decreased proliferative capacities induced by down-regulated DLG1-AS1 were elevated by the up-regulation of SSRP1 (Fig. 5b, c). The protein levels of PCNA, CDK1 and Cyclin D1 decreased by DLG1-AS1 knockdown were enhanced again by the overexpression of SSRP1 (Additional file 9: Fig. S9a). On the contrary, silenced DLG1-AS-induced apoptosis was recovered by SSRP1 overexpression (Fig. 5d, e and Additional file 9: Fig. S9b). Moreover, the migration and invasion suppressed by silenced DLG1-AS1 were rescued by the strengthened SSRP1 (Fig. 5f, g). The signals of AKT/mTOR and Src/FAK pathways weakened by DLG1-AS1 silencing were enhanced again by the up-regulation of SSRP1 (Additional file 9: Fig. S9c, d). In addition, the effects of SSRP1 overexpression on HCC cellular functions were also detected. It was uncovered that upregulation of SSRP1 facilitated HCC cell proliferation, migration and invasion (Additional file 10: Fig. S10a–d). In a word, DLG1-AS1 could boost HCC progression via enhancing SSRP1 expression.

### Silencing of DLG1-AS1 inhibits HCC cell growth in vivo

**Fig. 6:**
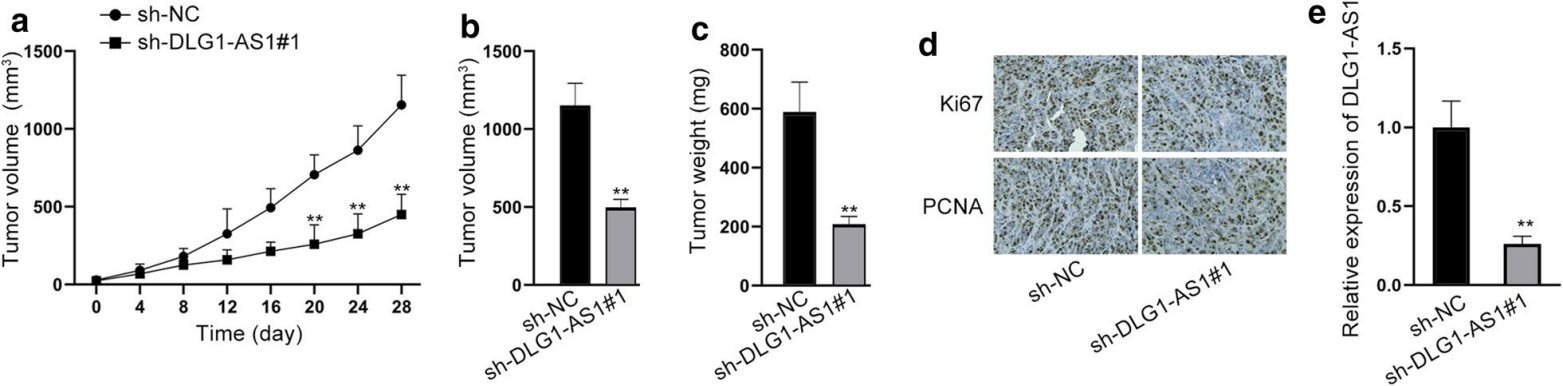
Silencing of DLG1-AS1 inhibits HCC cell growth in vivo. **a** Tumor growth curve was shown in sh-NC and sh-DLG1-AS1#1 group. **b**, **c** Tumor volume and weight as the last day were measured. **d** IHC detected Ki67 and PCNA in sh-NC and sh-DLG1-AS1#1 groups. **e** The level of DLG1-AS1 in tumors derived from HCCLM3 cells stably transfected with sh-NC or sh-DLG1-AS1#1. ^**^P < 0.01

We further used in vivo experiments to certify the results of in vitro experiments. HCCLM3 cells transfected with sh-NC and sh-DLG1-AS1#1 were transplanted into mice separately. After 28 days, all the mice were killed. The volume and weight of tumors were smaller in sh-DLG1-AS1#1 group than that in sh-NC group (Fig. 6a–c). According to IHC results, Ki67 and PCNA proteins were decreased by DLG1-AS1 silencing (Fig. 6d). The level of DLG1-AS1 was detected in two groups. As expected, the level of DLG1-AS1 was lower in sh-DLG1-AS1#1 group compared to sh-NC group (Fig. 6e). To sum up, knockdown of DLG1-AS1 could slow up the growth of HCC cells in vivo.

## Discussion

The distant metastasis occurs in patients with advanced HCC, which lead to high recurrence rate [27]. Exploring novel biomarkers in HCC is essential for improving early diagnosis. A host of lncRNAs can transform from transcriptional noise into crucial regulators in various diseases [28]. LncRNAs have been identified to have fundamental functions in cancers [29]. For example, HULC facilitates HCC progression by stabilizing COX-2 protein [30]. DANCR enhances stemness of HCC via up-regulating CTNNB1 [31]. In the present study, we discovered a highly expressed lncRNA DLG1-AS1 in HCC cells. Through functional assays, we determined that DLG1-AS1 silencing hampered cell proliferation, migration and invasion but promoted apoptosis in HCC. The results of in vivo experiments validated the role of DLG1-AS1 in promoting HCC tumor growth. To our knowledge, it is the first time to explore the expression and function of DLG1-AS1 in HCC cells.

LncRNAs can exert oncogenic functions through activating various signaling pathways [32–35]. In our current study, we found that DLG1-AS1 could activate PI3K/AKT and Src/FAK pathways and thus promoted HCC progression. This is also the first time to unveil the association between DLG1-AS1 and PI3K/AKT and Src/FAK pathways.

Upregulation of lncRNAs in human cancers may be induced by transcriptional activation [13–16]. MYC has been reported to be a transcriptional activator for lncRNAs in human cancers [36–38]. In this study, we demonstrated that MYC could activate the transcription of DLG1-AS1. Besides, we found that MYC could accelerate the progression of HCC via up-regulating DLG1-AS1. Importantly, we also uncovered that DLG1-AS1 could regulate MYC expression in HCC cells. Functionally, MYC/DLG1-AS1 axis could promote HCC cell growth and migration through activating PI3K/AKT and Src/FAK pathways.

CeRNA regulatory system has been widely reported in human cancers. For instance, LINC01133 functions as a ceRNA in gastric cancer by sponging miR-106a-3p to liberate APC [39]. LncRNA PTAR promotes the EMT and metastasis in ovarian cancer by acting as a ceRNA to targeting miR-101-3p/ZEB1 axis [40]. In our research, we also determined the ceRNA feature of DLG1-AS1 in HCC. Through mechanism investigation, we determined that miR-497-5p could interact with DLG1-AS1. The latest researches have demonstrated the tumor suppressive role of miR-497-5p in HCC [41, 42]. Here, we also identified that miR-497-5p had suppressive effects on HCC cell growth and migration.

In previous studies, SSRP1 was proven to exert oncogenic function in colorectal cancer [43], glioma [44] and HCC [45]. In our current study, we uncovered that SSRP1 was the downstream target of miR-497-5p and positively regulated by DLG1-AS1 in HCC cells. Importantly, SSRP1 involved in DLG1-AS1-mediated HCC cell functions. Thus, we confirmed that DLG1-AS1 enhanced SSRP1 level and induced HCC cellular processes by sponging miR-497-5p.

In summary, our research firstly found that MYC-induced upregulation of DLG1-AS1 boosts HCC cell growth and migration by regulating miR-497-5p/SSRP1 axis, suggesting the potential role of DLG1-AS1 as therapeutic target for HCC. However, lack of clinical study is a shortcoming of our current study. Thus, we will unmask the clinical significance of this novel molecular pathway in our future study. The specific mechanism by which DLG1-AS1 regulated MYC remains to be investigated in our future study.

## Conclusions

Our study firstly revealed the role of DLG1-AS1 in HCC cell functions. MYC-induced the transcriptional activation of DLG1-AS1 in HCC cells and promoted HCC cell growth and migration. More importantly, DLG1-AS1 elevated the expression level of SSRP1 by sponging miR-497-5p in HCC cells. Our findings might provide a novel therapeutic target for HCC patients.

## Supplementary Information

**Additional file 1: Figure S1.** The effects of DLG1-AS1 silencing on functional proteins.

**Additional file 2: Figure S2. **DLG1-AS1 silencing inactivates AKT/mTOR and Src/FAK signaling pathways.

**Additional file 3: Figure S3.** Overexpression of DLG1-AS1 facilitates THLE-2 cell proliferation, migration and invasion.

**Additional file 4: Figure S4. **The regulatory effect of DLG1-AS1 on MYC expression.

**Additional file 5: Figure S5. **MYC regulates AKT/mTOR and Src/FAK signaling pathways through DLG1-AS1.

**Additional file 6: Figure S6.** DLG1-AS1 promotes HCC progression and activates AKT/mTOR and Src/FAK signaling pathways through upregulating MYC.

**Additional file 7: Figure S7. **DLG1-AS1 modulates AKT/mTOR and Src/FAK signaling pathways through miR-497-5p.

**Additional file 8: Figure S8. **Inhibition of miR-497-5p promotes HCC cell proliferation, migration and invasion.

**Additional file 9: Figure S9. **DLG1-AS1 activates AKT/mTOR and Src/FAK signaling pathways by enhancing SSRP1 level.

**Additional file 10: Figure S10. **Overexpression of SSRP1 facilitates HCC cell proliferation, migration and invasion.

## Data Availability

Research and data material are not shared.

## Abbreviations

lncRNAs: Long non-coding RNAs
ceRNA: Competing endogenous RNA
miRNAs: MicroRNAs
DLG1-AS: DLG1 antisense RNA 1
SSRP1: Structure specific recognition protein 1
EMT: Epithelial mesenchymal transition
RIP: RNA immunoprecipitation
IHC: Immunohistochemistry
HCC: Hepatocellular carcinoma
ChIP: Chromatin immunoprecipitation
