# Supplementary figures and images for: DLG1-AS1 is activated by MYC and drives the proliferation and migration of hepatocellular carcinoma cells through miR-497-5p/SSRP1 axis
Source: Cancer Cell Int. 2021 Jan 6;21:16. doi: 10.1186/s12935-020-01667-0 (PMC7789637; doi:10.1186/s12935-020-01667-0)

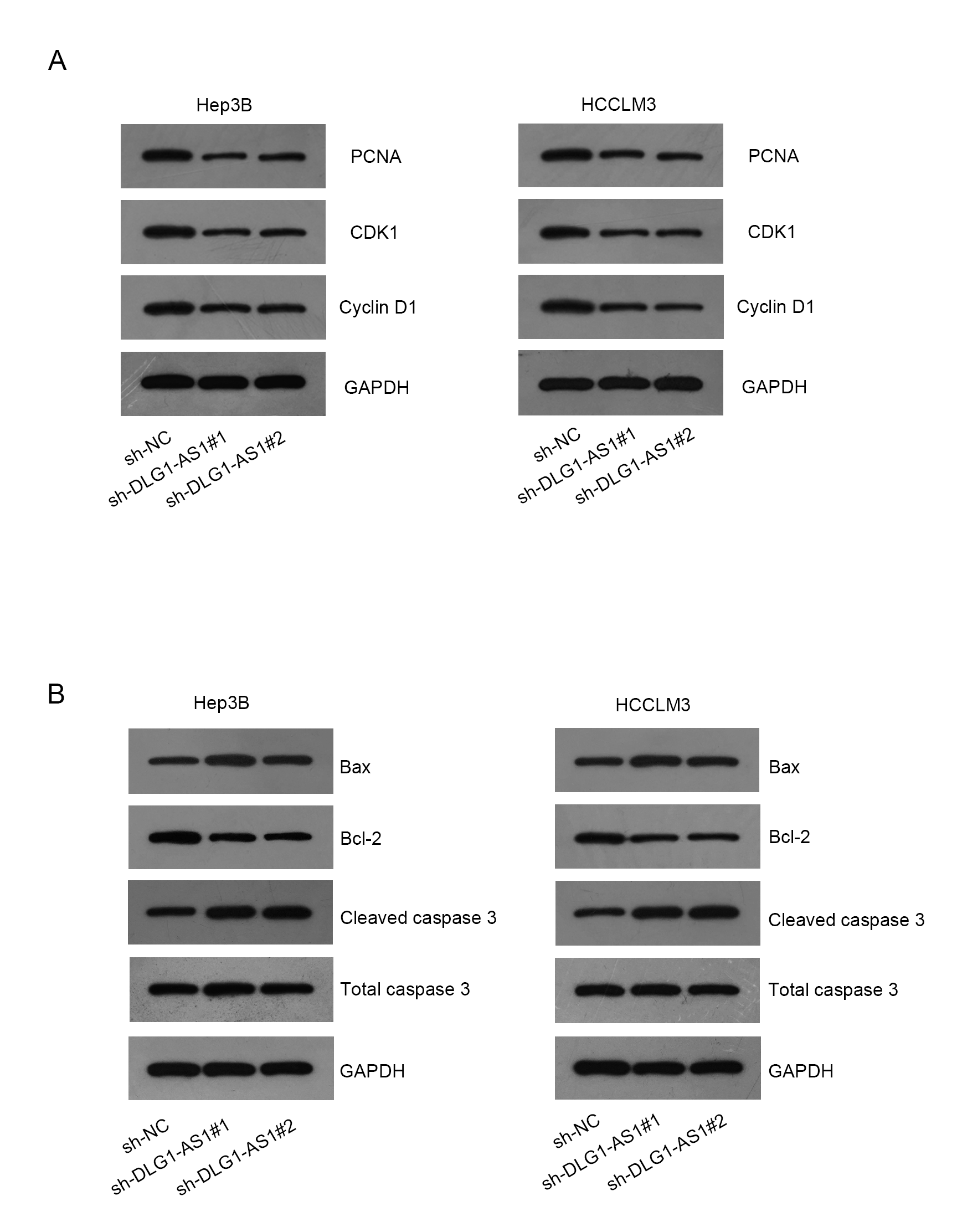

Supplement: Supplementary file 1 — Additional file 1: Figure S1. The effects of DLG1-AS1 silencing on functional proteins. [file 12935_2020_1667_MOESM1_ESM.tif]

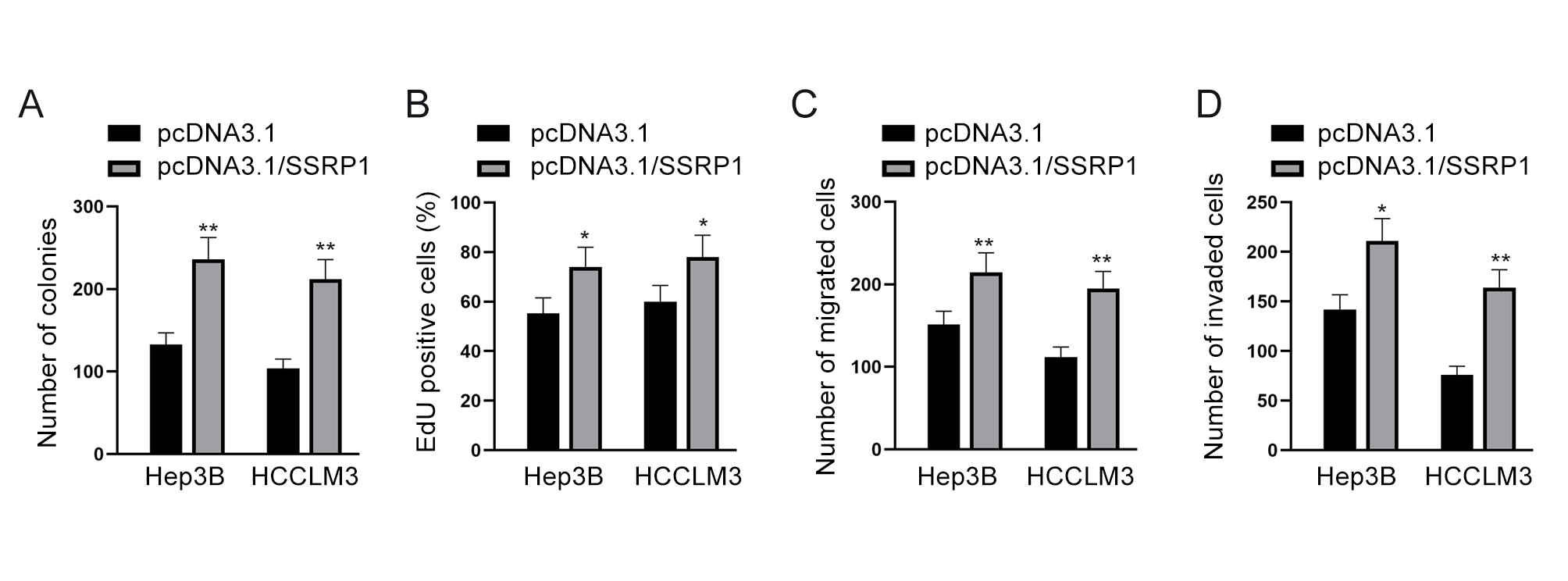

Supplement: Supplementary file 2 — Additional file 2: Figure S2. DLG1-AS1 silencing inactivates AKT/mTOR and Src/FAK signaling pathways. [file 12935_2020_1667_MOESM2_ESM.tif]

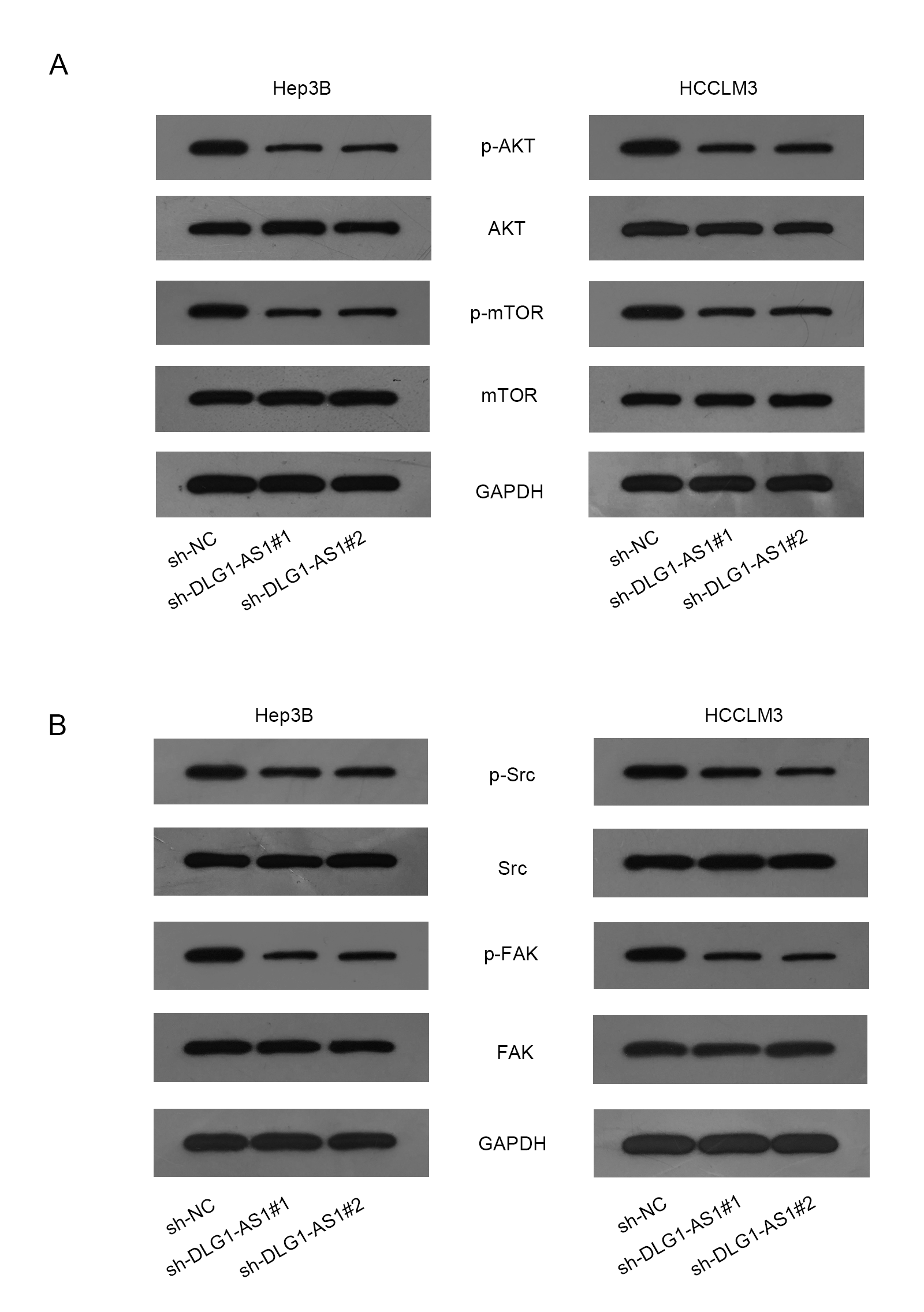

Supplement: Supplementary file 3 — Additional file 3: Figure S3. Overexpression of DLG1-AS1 facilitates THLE-2 cell proliferation, migration and invasion. [file 12935_2020_1667_MOESM3_ESM.tif]

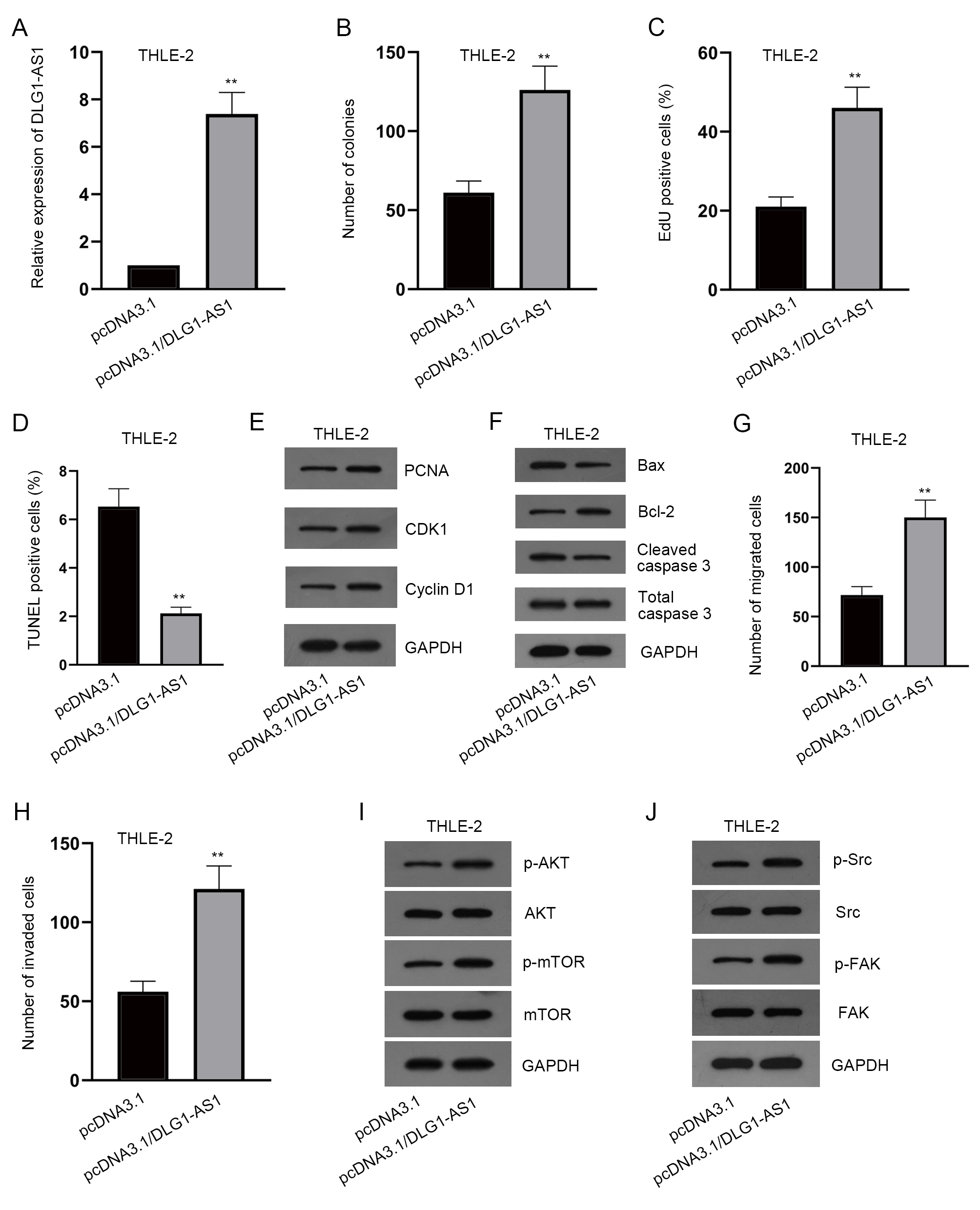

Supplement: Supplementary file 4 — Additional file 4: Figure S4. The regulatory effect of DLG1-AS1 on MYC expression. [file 12935_2020_1667_MOESM4_ESM.tif]

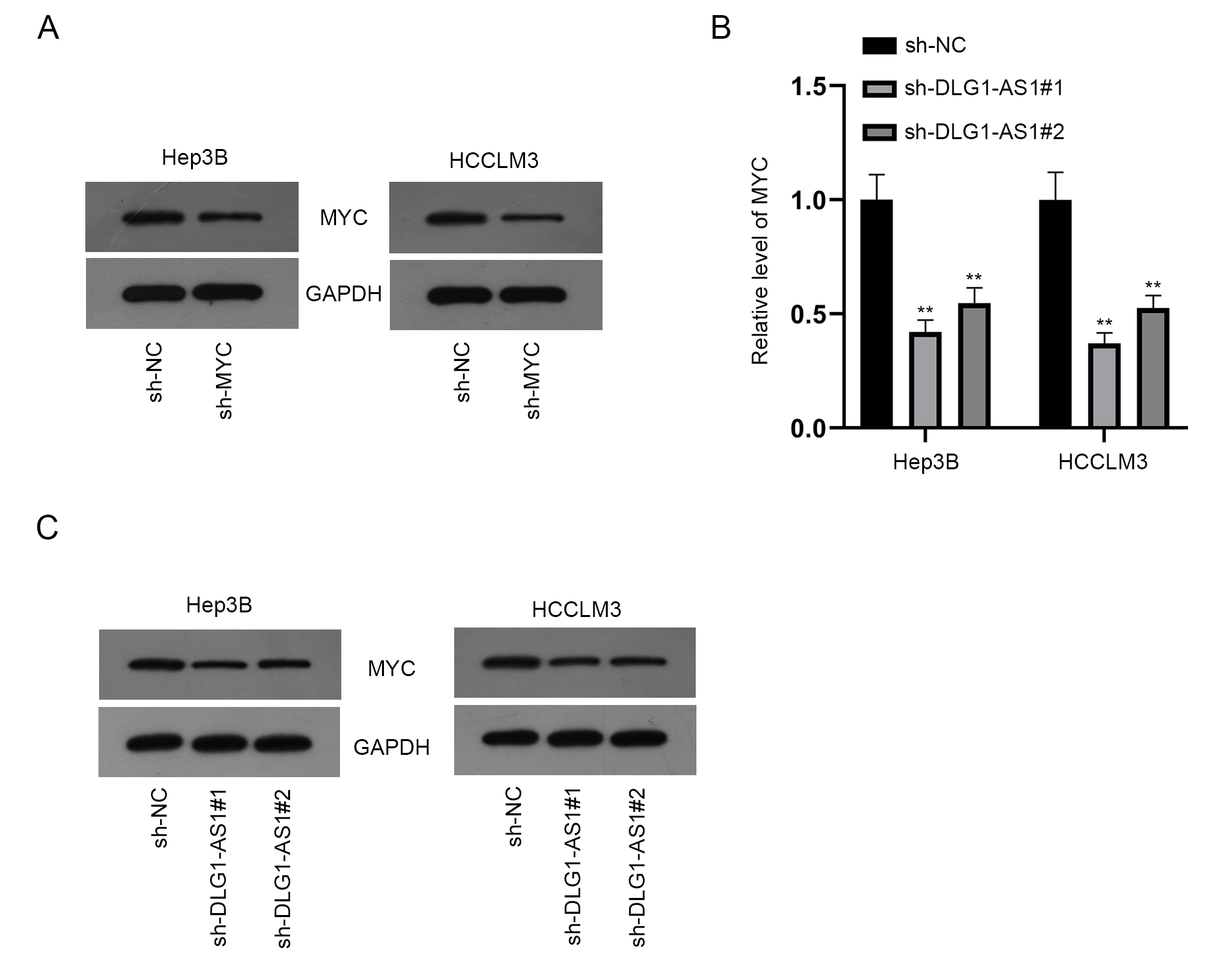

Supplement: Supplementary file 5 — Additional file 5: Figure S5. MYC regulates AKT/mTOR and Src/FAK signaling pathways through DLG1-AS1. [file 12935_2020_1667_MOESM5_ESM.tif]

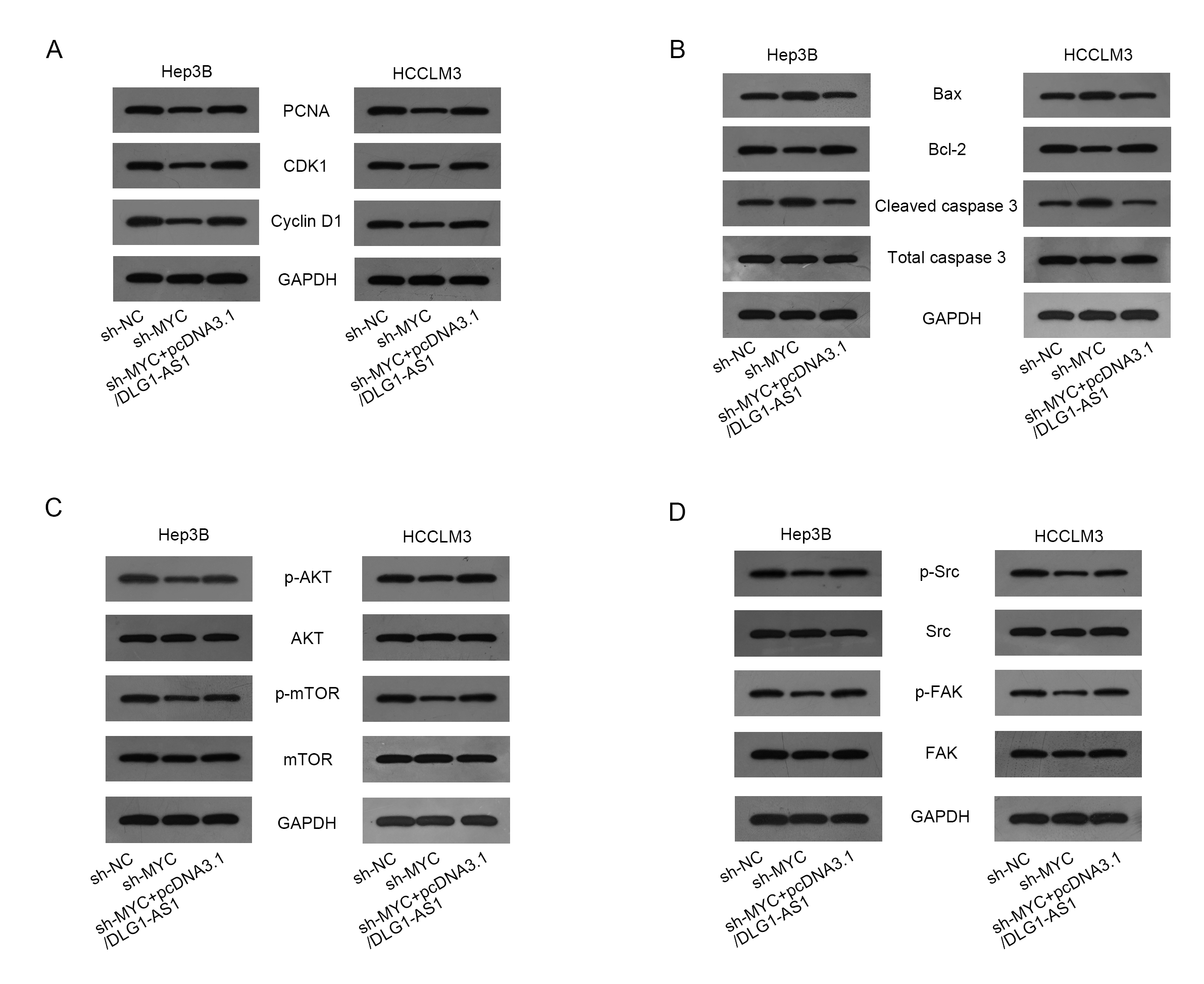

Supplement: Supplementary file 6 — Additional file 6: Figure S6. DLG1-AS1 promotes HCC progression and activates AKT/mTOR and Src/FAK signaling pathways through upregulating MYC. [file 12935_2020_1667_MOESM6_ESM.tif]

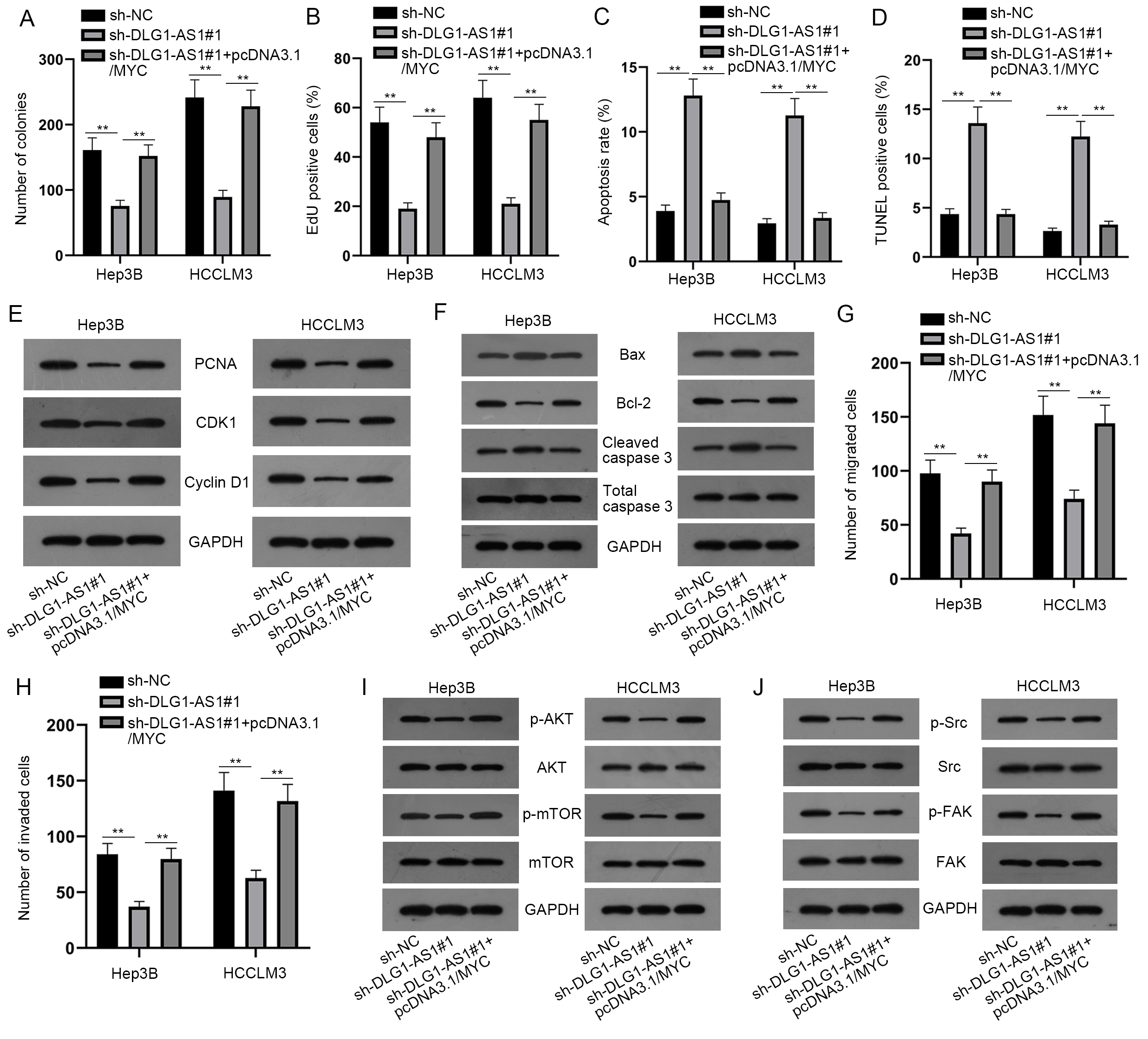

Supplement: Supplementary file 7 — Additional file 7: Figure S7. DLG1-AS1 modulates AKT/mTOR and Src/FAK signaling pathways through miR-497-5p. [file 12935_2020_1667_MOESM7_ESM.tif]

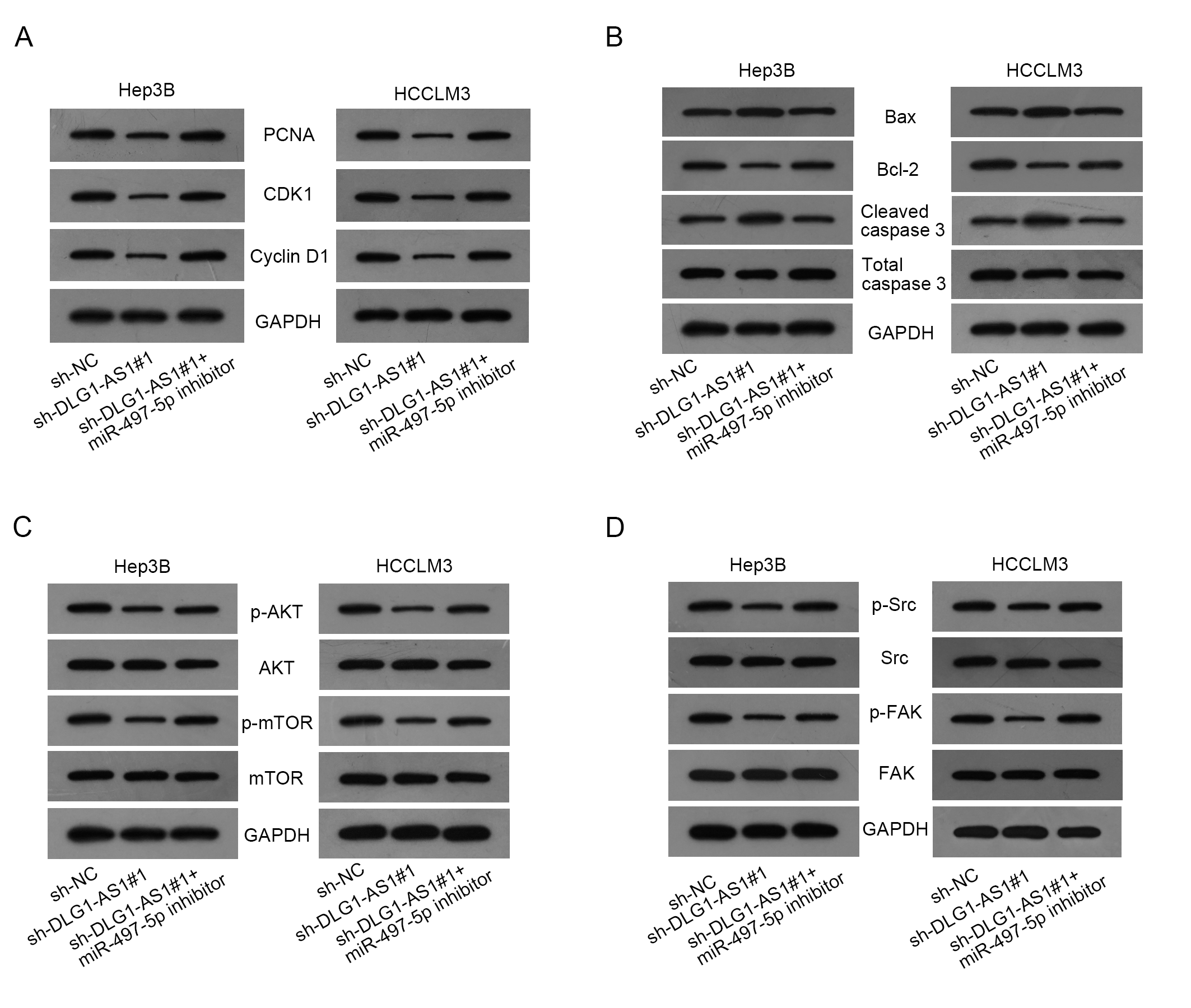

Supplement: Supplementary file 8 — Additional file 8: Figure S8. Inhibition of miR-497-5p promotes HCC cell proliferation, migration and invasion. [file 12935_2020_1667_MOESM8_ESM.tif]

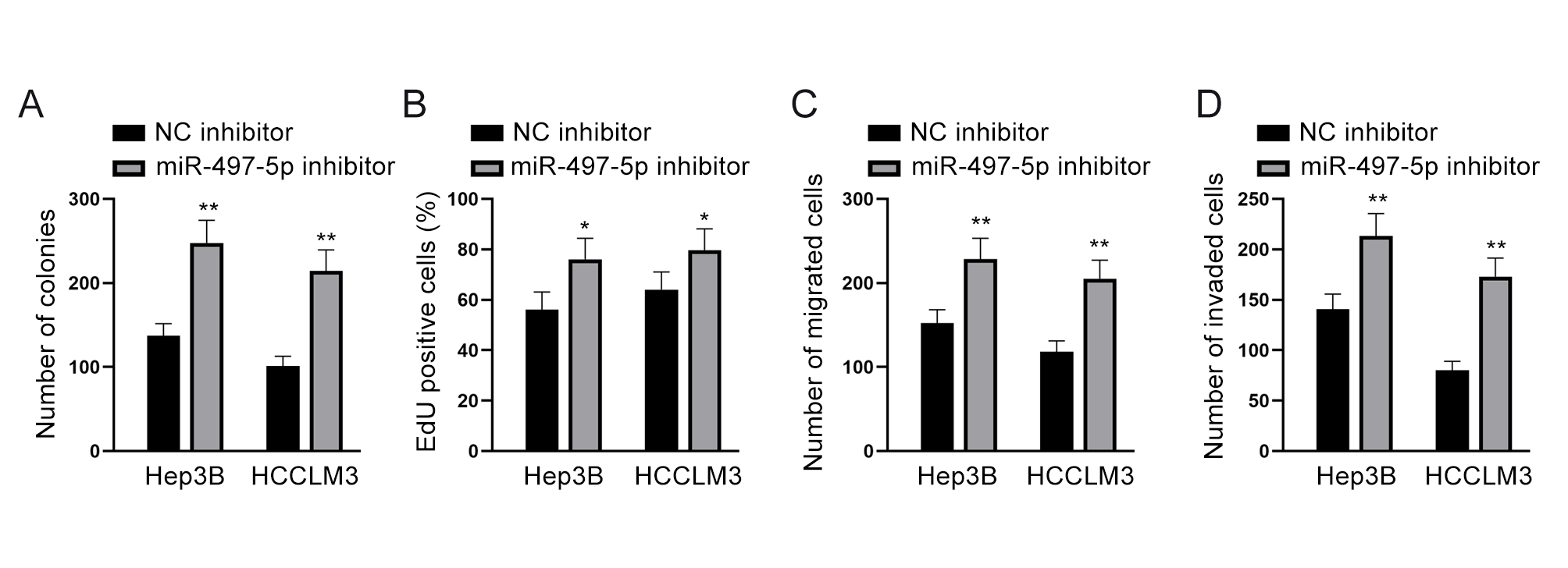

Supplement: Supplementary file 9 — Additional file 9: Figure S9. DLG1-AS1 activates AKT/mTOR and Src/FAK signaling pathways by enhancing SSRP1 level. [file 12935_2020_1667_MOESM9_ESM.tif]

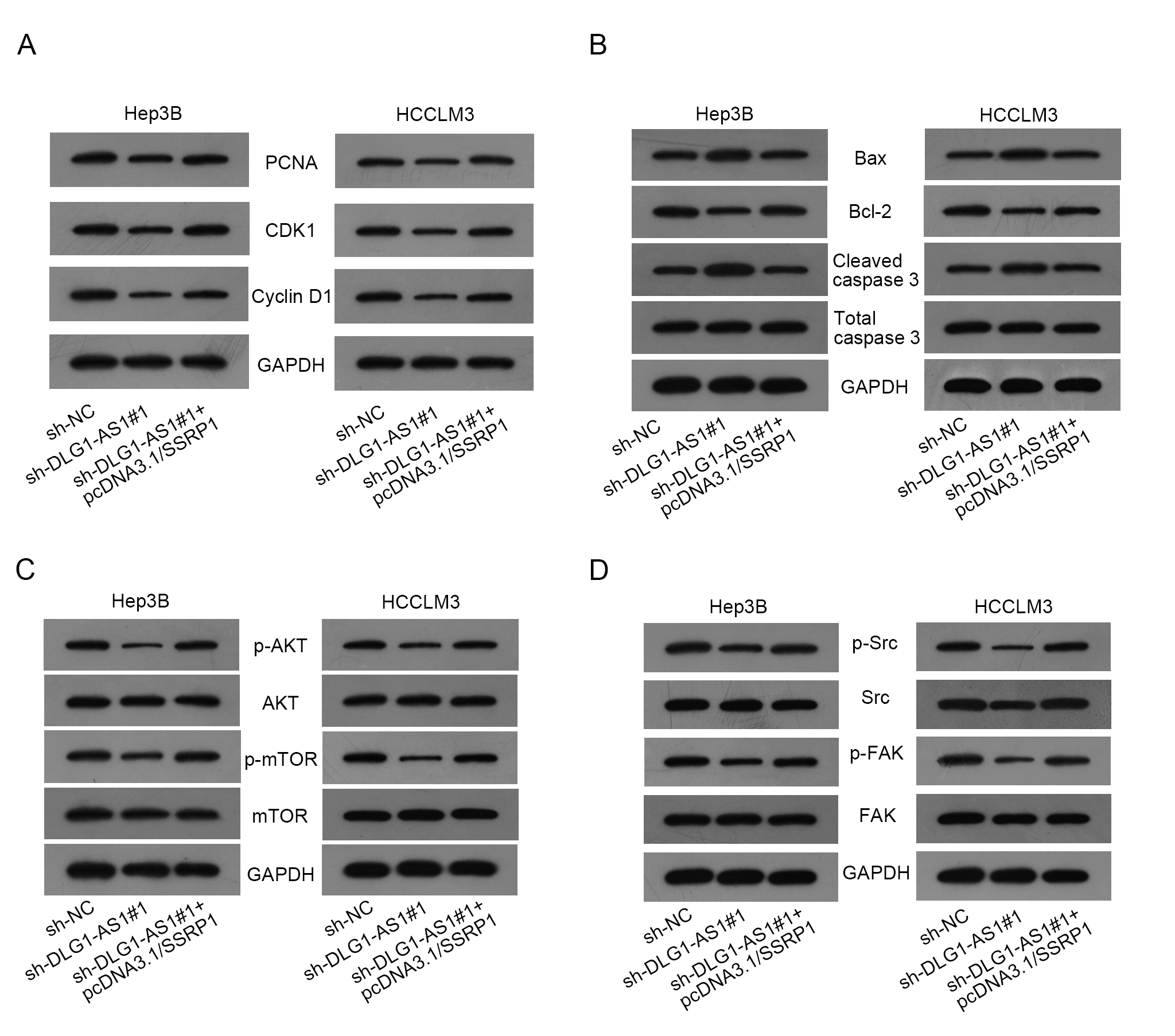

Supplement: Supplementary file 10 — Additional file 10: Figure S10. Overexpression of SSRP1 facilitates HCC cell proliferation, migration and invasion. [file 12935_2020_1667_MOESM10_ESM.tif]
